# Targeted DNA demethylation of the *Arabidopsis* genome using the SunTag-dCpf1-TET1cd system

**DOI:** 10.17912/micropub.biology.000814

**Published:** 2023-03-20

**Authors:** Yunxi Zheng, Reqing He

**Affiliations:** 1 Queen Mary School, Nanchang University, Nanchang 330031, China; 2 Key Laboratory of Molecular Biology and Gene Engineering in Jiangxi Province, College of Life Science, Nanchang University,Nanchang 330031, China

## Abstract

DNA methylation is a stable and heritable epigenetic mark, and it plays an important role in regulation of gene expression and transposon silencing. Here we developed a CRISPR/dCpf1-based targeted demethylation system using the catalytic domain of the human demethylase TEN-ELEVEN TRANSLOCATION1 (TET1cd) and a SunTag system. The SunTag-dCpf1-TET1cd system is able to achieve targeted DNA demethylation and up-regulate gene expression when guided to the *FWA* or *CACTA1* loci in *Arabidopsis* *thaliana* . Our study provides tools for targeted removal of DNA cytosine methylation, and activation of protein-coding genes or transposons expression.

**Figure 1. Targeted demethylation of FWA and CACTA1 using SunTag-dAsCpf1/dLbCpf1-TET1cd system in Arabidopsis .**
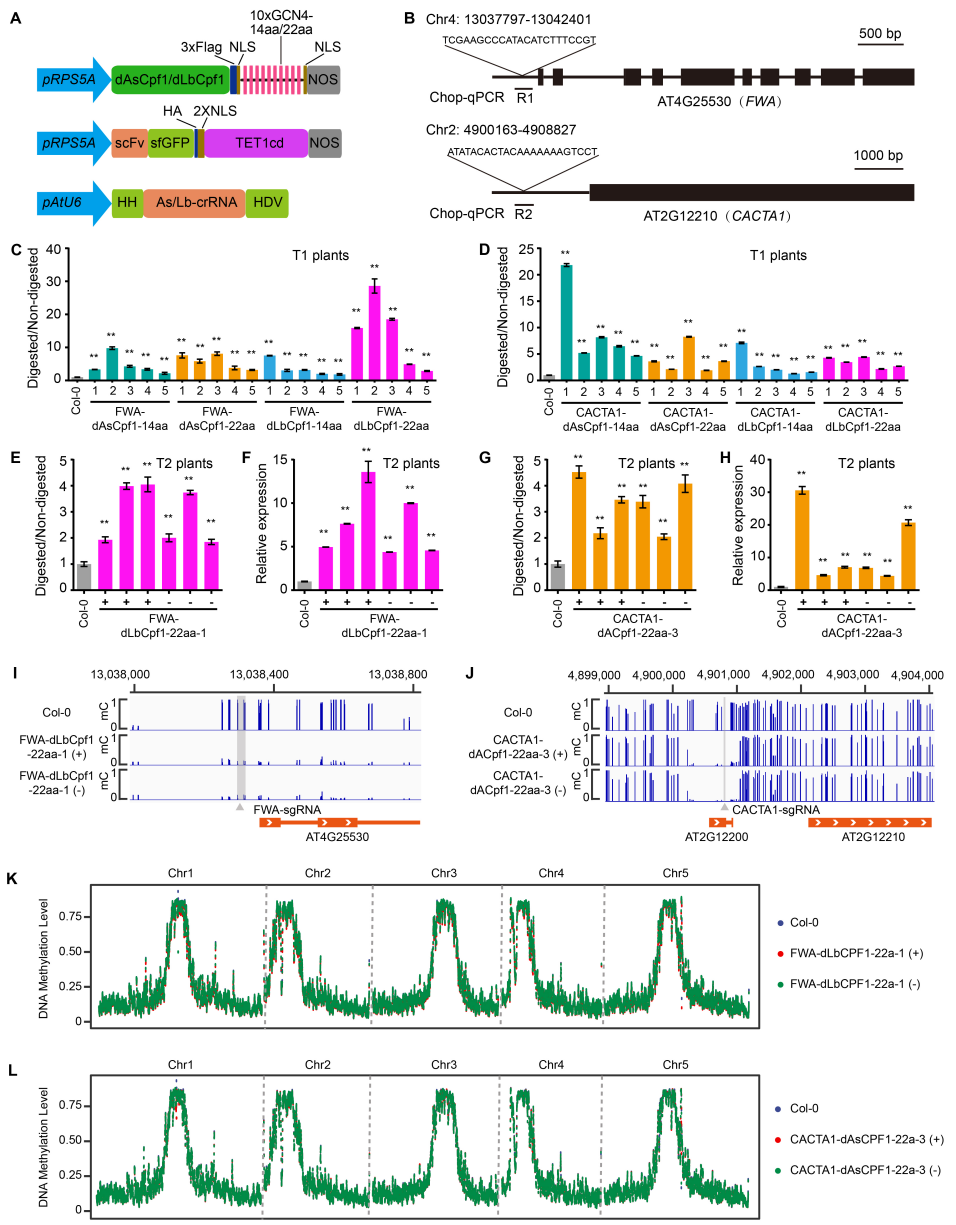
(A) Schematic representation of the SunTag-dAsCpf1/dLbCpf1-TET1cd system. pRPS5A: *Arabidopsis* *thaliana* RPS5A promoter, dAsCpf1/dLbCpf1: nuclease-inactive AsCpf1/LbCpf1, aa: amino acids, NLS: nuclear localization signal, GCN4: yeast transcription factor GCN4, scFv: single chain antibody fragment, sfGFP: superfolder-GFP, TET1cd: catalytic domain of the human demethylase Ten-Eleven Translocation 1 (TET1), pAtU6: Arabidopsis thaliana U6 promoter, crRNA: CRISPR RNA, HH: hammerhead ribozyme, HDV: hepatitis delta virus ribozyme. (B) Schematic view of structures of *FWA* and *CACTA1* loci. The target sites, and the regions for McrBC digestion followed by real-time PCR (Chop-qPCR) analysis (R1 and R2) are shown. Boxes and lines indicate exon and intron, respectively. (C) Chop-qPCR assays showing the methylation levels in the *FWA* promoter region for Col-0, FWA-dAsCpf1-14aa, FWA-dAsCpf1-22aa, FWA-dLbCpf1-14aa and FWA-dLbCpf1-22aa T1 plants. *TUB2* is used as an internal control. (D) Chop-qPCR assays showing the methylation levels in the *CACTA1* promoter region for Col-0, CACTA1-dAsCpf1-14aa, CACTA1-dAsCpf1-22aa, CACTA1-dLbCpf1-14aa and CACTA1-dLbCpf1-22aa T1 plants. (E) Chop-qPCR assays showing the methylation levels in the *FWA* promoter region for Col-0 and six representative FWA-dLbCpf1-22aa T2 plants containing the transgene (+) or that had segregated it away (-). (F) Expression analysis of the *FWA* in Col-0 and on representative FWA-dLbCpf1-22aa T2 line. *IPP2* is used as an internal control. (G) Chop-qPCR assays showing the methylation levels in the *CACTA1* promoter region for Col-0 and six representative CACTA1-dAsCpf1-22aa T2 plants containing the transgene (+) or that had segregated it away (-). (H) Expression analysis of the *CACTA1* in Col-0 and one representative CACTA1-dAsCpf1-22aa T2 line. (I) Screenshot of DNA methylation status of the *FWA* target region in Col-0, one representative T2 plant of FWA-dLbCPF1-22a-1 line containing the transgene (+) or that had segregated it away (-). A gray vertical line indicates the FWA-sgRNA binding site. (J) Screenshot of DNA methylation status of the *CACTA1* target region in Col-0, one representative T2 plant of CACTA1-dAsCPF1-22a-3 line containing the transgene (+) or that had segregated it away (-). A gray vertical line indicates the CACTA1-sgRNA binding site. (K) Genome-wide distribution of cytosine DNA methylation in Col-0, one representative T2 plant of FWA-dLbCPF1-22a-1 line containing the transgene (+) or that had segregated it away (-). (L) Genome-wide distribution of cytosine DNA methylation in Col-0, one representative T2 plant of CACTA1-dAsCPF1-22a-3 line containing the transgene (+) or that had segregated it away (-). Data shown as mean ± SD (n = 3 technical replicates). *FWA* : AT4G25530; *CACTA1* : AT2G12210; *IPP2* : AT3G02780; *TUB2* : AT5G62690. Values shown are means ± SD from three replicates. Asterisks represent significant differences determined by Student’s *t* -test (** *P* < 0.01).

## Description

DNA methylation as an important epigenetic mark is crucial for diverse biological processes in many higher eukaryotes, and it occurs in three sequence contexts including CG, CHG and CHH (H = A, T or C) in plants (Law and Jacobsen, 2010). Plant DNA methylation patterns are stably inherited over generations (Becker et al., 2011; Schmitz et al., 2011), and differences in DNA methylation can lead to the formation of epialleles. Epialleles have been found to associate with phenotypic traits, such as a late-flowering phenotype of the *fwa-4* epiallele that is caused by loss of DNA methylation in the promoter of the *FLOWERING WAGENINGEN* ( *FWA,* AT4G25530) gene (Johnson et al., 2014), which provides a new genetic source for crop breeding. CRISPR/dCas9-based SunTag-TET1cd system has been recently applied to achieve targeted DNA demethylation and up-regulation of the target gene, such as *FWA* and *CACTA1* (AT2G12210) in Arabidopsis (Gallego-Bartolome et al., 2018), and *FIE1* and *Tos17* rice (Tang et al., 2021). In that system, the target site specificity of CRISPR/SpCas9 is strictly determined by a single guide RNA (sgRNA) and a NGG protospacer adjacent motif (PAM) in the genome. Cpf1, an endonuclease of the class 2 CRISPR family, employs the TTTN PAM (Zetsche et al., 2015), complementing the SpCas9 system. Here, we developed the SunTag-dCpf1-TET1cd system, to produce targeted DNA demethylation and up-regulation of *FWA* and *CACTA1* in *Arabidopsis* *thaliana* . Both dAsCpf1/dLbCpf1 and scFv modules were expressed under the control of the *A.* *thaliana RPS5A* promoter that maintains high constitute expression at all developmental stages starting from the egg cell and including meristematic cells (Tsutsui and Higashiyama, 2017), and the *U6* promoter of *A.* *thaliana* was applied to effectively express crRNA (Figure 1A). Two self-cleaving ribozymes, hammerhead ribozyme and hepatitis delta virus ribozyme, were used for processing sgRNA (Figure 1A). Two versions of the epitope tail fused to dAsCpf1/dLbCpf1 were produced, one harbouring a 14-aa linker, and the other harbouring a 22-aa linker (Figure 1A). The crRNA targeting the promoter region of *FWA* was designed (Figure 1B), and then the editing reagents were transformed into Col-0 plants for generating FWA-dAsCpf1-14aa, FWA-dAsCpf1-22aa, FWA-dLbCpf1-14aa and FWA-dLbCpf1-22aa transgenic plants. Methylation level of targeted region was examined by using Chop-qPCR, in which DNAs from 20 randomly selected T1 plants were digested with McrBC that only cuts at methylated sequences. All transgenic plants presented evident hypomethylation around the target site compared to Col-0, and SunTag-dCpf1-TET1cd system including dLbCpf1 and 22-a linker exhibited the best performance (Figure 1C). The heritability of targeted demethylation caused by SunTag-dCpf1-TET1cd system was further studied, we performed Chop-qPCR on six T2 plants from the same FWA-dLbCpf1-22aa transgenic line that had the transgene (+) or had segregated it away (-). Compared with Col-0, various degrees of hypomethylation of the targeted regions were observed in FWA-dLbCpf1-22aa T2 plants, and RT-qPCR results shown that expression level of *FWA* was clearly up-regulated in these T2 plants (Figure 1 E and F). Next, a heterochromatic locus, *CACTA1* was chosen to test the targeted demethylation by using our SunTag-dCpf1-TET1cd system. We examined methylation levels around this target site in 20 randomly selected T1 transgenic plants, and all T1 plants showed distinct hypomethylation on targeted region compared to Col-0, and SunTag-dCpf1-TET1cd system including dAsCpf1 and 14-aa linker exhibited the best performance (Figure 1D). The inheritance of targeted demethylation on *CACTA1* was also explored by using the CACTA1-dAsCpf1-22aa line 3, and various degrees of hypomethylation of the targeted regions were observed in its T2 plants that had the transgene (+) or had segregated it away (-) comparing to Col-0 (Figure 1G). Moreover, the remarkable up-regulation of *CACTA1* was found in these T2 plants (Figure 1H). In addition, we separately selected two independent T2 plants from FWA-dLbCPF1-22a-1 and CACTA1-dAsCPF1-22a-3 to perform whole-genome bisulfite sequencing. In both cases, we found efficient demethylation at target sites in T2 plants that had the transgene or had segregated it away (Figure 1 I and J), supporting that SunTag-dCpf1-TET1cd system could produce heritable demethylation at its targeted region, even in the absence of transgenes. Analysis of genome-wide methylation shown that the overall methylation levels of the edited plants were comparable to that of Col-0 (Figure 1 K and L). Taken together, our results demonstrate that SunTag-dCpf1-TET1cd system works effectively in Arabidopsis, which enriches targeted DNA demethylation tools in plants.

## Methods

**Plant growth and transformation**

*Arabidopsis* *thaliana* plants used in this study were under the Col-0 background, and were grown under long-day conditions (16 h light, 8 h dark cycle) at 22 °C. All produced plasmids were transformed into *Agrobacterium* *tumefaciens* strain GV3101, and then transformed into Col-0 plants *via* the flower dipping method.

**Plasmid construction**

The nuclease domains of AsCpf1 and LbCpf1 were deactivated by mutation according to their conservation (Zetsche et al., 2015), generating dAsCpf1 (D908A and E993A) and dLbCpf1 (D832A and E925A). To construct SunTag-dCpf1-TET1cd binary vector in Arabidopsis, the sequences of *RPS5A* promoter and dCpf1 were amplified, and used to replace *UBQ10* promoter and dCas9 of SunTag-22aa or SunTag-14aa (Gallego-Bartolome et al., 2018), respectively. After that, the crRNA scaffold with HH and HDV sequences were linked together by overlapping PCR, and then fused to the *AtU6* promoter by Gibson assembly (Gibson et al., 2009). Nucleotide sequences of SunTag-dLbCpf1-22a-TET1cd, SunTag-dAsCpf1-22a-TET1cd, SunTag-dLbCpf1-14aa-TET1cd and SunTag-dAsCpf1-14a-TET1cd are provided in Dataset S1.

**Chop-qPCR and RT-qPCR**

Chop-qPCR was performed as previously described (Liu et al., 2022). Briefly, CTAB-extracted DNA (1 µg) digested with McrBC at 37 °C overnight was applied to amplify the indicated regions. For RT-qPCR, total RNA was extracted using Trizol (Ambion), and then the first-strand complementary DNA was synthesized using TrasnScript One-Step gDNA Removal and cDNA Synthesis SuperMix (Transgen). Real-time PCR was executed applying SYBR qPCR Master Mix (Vazyme) on a CFX96 Real-Time PCR Detection System (Bio-Rad). Primers used in Chop-qPCR and RT-qPCR are listed in Table 1.

**Whole-genome bisulfite sequencing analysis**

Genomic DNAs were extracted from 2-week-old seedlings, and sent to Novogene company (Beijing, China) for whole-genome bisulfite sequencing. For bisulfite sequencing data processing, adaptor and low-quality reads were removed using bbduk.sh (v38.90) with following parameters: hdist=1 mink=11 ktrim=r qtrim=rl trimq=20 minlen=25 trimpolyg=20. Next, clean reads were mapped to the Col-0 TAIR10 *Arabidopsis* *thaliana* genome using Bismark (v0.19.1) with default settings (Krueger and Andrews, 2011), context-dependent methylations were identified and extracted using “bismark_methylation_extractor” of Bismark package. The Arabidopsis chromosomes were split into 10kb sliding windows with a stepping size of 1kb using BEDOPS (v2.4.40) (Neph et al., 2012). The methylation levels (measured by the averaged percentage of methylation of cytosines) for each sliding window were calculated using BEDOPS and visualized using ggplot2.

**Additional Information**

The whole-genome bisulfite sequencing data has been deposited at NCBI’s GEO database repository under the accession GSE221829.

## Reagents

**Table:** 

**Table 1.** Information for primers		
**Primer names**	**Sequences (5ˈ−>3ˈ)**	**Purpose**
*FWA* -qFP	TTAGATCCAAAGGAGTATCAAAG	RT-qPCR
*FWA* -qRP	CTTTGGTACCAGCGGAGA	RT-qPCR
*CACTA1* -qFP	AGTGTTTCAATCAAGGCGTTTC	RT-qPCR
*CACTA1* -qRP	CACCCAATGGAACAAAGTGAAC	RT-qPCR
*IPP2* - qFP	GTATGAGTTGCTTCTCCAGCAAAG	RT-qPCR
*IPP2* - qRP	GAGGATGGCTGCAACAAGTGT	RT-qPCR
*FWA* -FP	TTGGGTTTAGTGTTTACTTG	Chop-qPCR
*FWA* -RP	GAATGTTGAATGGGATAAGGTA	Chop-qPCR
*CACTA1* -FP	CGCAGTACTCATTCTCACATGAT	Chop-qPCR
*CACTA1* -RP	CATTCCCGCTAGAGGATTTACGG	Chop-qPCR
*TUB* 2-FP	CCGAGCACGGCATCGATCCAA	Chop-qPCR
T *UB* 2-RP	TGAGCACTGCACGAGGAACGA	Chop-qPCR
